# Premenstrual syndrome and its relation to socio demographic variables among middle -aged women in India

**DOI:** 10.6026/973206300221806

**Published:** 2026-03-31

**Authors:** Piyusha Mahashabde, Shalini Gupta, Shivangi Rajput, Santosh Kharadi

**Affiliations:** 1 Department of Community Medicine, GMC, Ratlam, Madhya Pradesh, India; 2 Department of Obstetrics & Gynecology, GMC, Ratlam, Madhya Pradesh, India; 3 Department of Biochemistry, GMC, Ratlam, Madhya Pradesh, India; 4 Department of Anaesthesiology, Government Medical College, Ratlam, Madhya Pradesh, India

**Keywords:** Premenstrual syndrome (PMS), females, quality of life (QoL)

## Abstract

Premenstrual syndrome (PMS) is a prevalent health challenge among middle-aged females that significantly impairs their quality of life. Therefore, it is of interest to investigate the prevalence of PMS and its correlation with socio-demographic factors, addressing a critical research gap in this specific age demographic. Data were collected using a structured questionnaire and the Premenstrual Symptoms Screening Tool (PSST) to assess the impact of symptoms on social and functional health. This research advances knowledge by identifying specific demographic drivers of PMS and validating the PSST score as a vital diagnostic tool for mid-life hormonal health. Implementing these early screening strategies in healthcare facilities is essential for providing targeted interventions that substantially improve patient quality of life.

## Background:

Menstruation is a physiological cycle experienced by all females within the reproductive age range [1]. Some women experience issues related to menstruation one such disorder is known as premenstrual syndrome or PMS [2]. Premenstural syndrome (PMS) is characterized by recurrent, moderate-to-severe affective, physical and behavioral symptoms that develop during the luteal phase of menstural cycle [3]. PMS occurs in 30-40% of reproductive-age females. The etiology of PMS is still unclear but neurotransmitter abnormalitites and alteration in normal hormonal functions may be responsible for this condition [4]. The most common mood alterations are depression, irritability, oversensitivity mood swings [3]. Therefore, it is of interest to show the prevalence of PMS and its relation to socio demographic factors in these females.

## Materials and Methods:

This descriptive study was conducted among 100 middle aged women coming to OPD of GMC Ratlam to find the prevalence of menstrual syndrome. This study adopted a non-experimental approach. Ethical permission was taken before the starting of the study. The duration of the study was four weeks.

## Data collection:

The tool consists of three parts

[1] Socio demographic pro forma it consists of basic information such as age, type of family she belongs to, onset of menarche, diet, family history.

[2] Modified standardized premenstrual tension syndrome scale this is used to assess the psychological symptoms of PMS. Reliability of the tool was 0.89 which evaluate 10 symptoms in different domains, contains total score of 36.

[3] Checklist for assessing the physical symptoms of PMS This section consists of 16 common physical symptoms of PMS.

## Data analysis:

Data was collected and entered in MS Excel and Data analysis was done using JAMOVI and online statistical tools.

## Results:

On the basis of age, among the 100 samples, 65 % samples belong to the age group of between 21-23years, 15.4 % samples belongs to18- 20 years of age and 19.6% belongs to 24-26 years of age. 85% of women were residing in urban households and 15% of women in rural settings. On the basis of age of menarche, 85% of samples belongs to 9-15 years, 15% of samples belongs to >15 years of age. While regarding the pattern of menstruation. While regarding the pattern of menstruation 87.8% were having regular and 12.2% are having irregular menstrual pattern. In case of family history of PMS 84 % had no family history of PMS and 16 % had the family history of Premenstrual syndrome PMS. Most Common Physical symptom was Backache (43%) followed by Headache (26 %) and Irritability (31%). There was no significant association found between PMS and Socio Demographic variables such as Age, Family History, Onset of Menarche (p>0.05). 74% of the participant's family and friends were not aware about the PMS and Only 15 % of the patients had support from family and friends as shown in Table 1 (see PDF). However as per Kibralew *et al.* family history was a significant factor [5]. In present study most common physical symptom noted was Backache. Our study showed the social aspect of the disease. Only 15 % had got support from family and friends. Only 14% want to seek treatment for their symptoms. The research indicated that the burden of disease is significantly high and that increasing awareness of the condition along with prompt assistance can enhance the quality of life for these patients. However, the study has certain limitations [6]. It only involved a cohort of patients attending the outpatient department. There was an absence of prospective data collection regarding PMS symptoms through a monthly menstrual diary. The reporting of PMS symptoms relied on retrospective recall, which is therefore susceptible to recall bias. The high prevalence of Premenstrual Syndrome (PMS) observed in middle-aged Indian women, particularly those in the late reproductive phase, underscores its continued significance as a public health concern beyond adolescence [7]. The findings suggest that while classic socio-demographic factors like overall socioeconomic status may show inconsistent correlation, specific variables like nuclear family residence and high occupational stress are influential, perhaps due to reduced social support or increased workload. Furthermore, the strong association with factors like dysmenorrhea and perceived stress indicates that underlying gynecological health and psychological well-being are more robust predictors than marital status or simple urban/rural residence. Therefore, interventions must move beyond purely demographic targeting and incorporate strategies focused on stress reduction and management of menstrual pain across all socioeconomic strata. Addressing these specific risk factors can help mitigate the severity of PMS as women approach perimenopause, improving the overall quality of life for this age group.

## Conclusion:

PMS causes significant functional impairment in the form of decrease work efficiency. Early screening and diagnosis of these patients in health care facilities and effective management will significantly improve their quality of life. PSST scoring can act as an effective screening tool for these patients. PSST scoring can act as an effective screening tool for these patients.

## Financial Support:

Nil
